# Digital Gene Expression Profiling Analysis of Aged Mice under Moxibustion Treatment

**DOI:** 10.1155/2018/4767328

**Published:** 2018-05-02

**Authors:** Nan Liu, Yunyao Jiang, Min Xing, Baixiao Zhao, Jincai Hou, Minyee Lim, Jian Huang, Xue Luo, Li Han

**Affiliations:** ^1^School of Clinical Medicine, Beijing University of Chinese Medicine, Beijing 10029, China; ^2^Institute of Basic Medical Sciences, Xiyuan Hospital, China Academy of Chinese Medical Sciences, Beijing 100091, China; ^3^Jing-Jin-Ji Joint Innovation Pharmaceutical (Beijing) Co., Ltd., Beijing 100083, China; ^4^School of Traditional Chinese Medicine, Beijing University of Chinese Medicine, Beijing 100029, China; ^5^School of Acupuncture-Moxibustion and Tuina, Beijing University of Chinese Medicine, Beijing 100029, China; ^6^International School, Beijing University of Chinese Medicine, Beijing 100029, China; ^7^Beijing Dongfang Hospital, Beijing University of Chinese Medicine, Beijing 100029, China

## Abstract

Aging is closely connected with death, progressive physiological decline, and increased risk of diseases, such as cancer, arteriosclerosis, heart disease, hypertension, and neurodegenerative diseases. It is reported that moxibustion can treat more than 300 kinds of diseases including aging related problems and can improve immune function and physiological functions. The digital gene expression profiling of aged mice with or without moxibustion treatment was investigated and the mechanisms of moxibustion in aged mice were speculated by gene ontology and pathway analysis in the study. Almost 145 million raw reads were obtained by digital gene expression analysis and about 140 million (96.55%) were clean reads. Five differentially expressed genes with an adjusted *P* value < 0.05 and |log⁡2(fold  change)| > 1 were identified between the control and moxibustion groups. They were Gm6563, Gm8116, Rps26-ps1, Nat8f4, and Igkv3-12. Gene ontology analysis was carried out by the GOseq R package and functional annotations of the differentially expressed genes related to translation, mRNA export from nucleus, mRNA transport, nuclear body, acetyltransferase activity, and so on. Kyoto Encyclopedia of Genes and Genomes database was used for pathway analysis and ribosome was the most significantly enriched pathway term.

## 1. Introduction

Aging is closely connected with death, progressive physiological decline, and increased risk of diseases, such as cancer, type 2 diabetes mellitus, all forms of arteriosclerosis, heart disease, hypertension, age-related macular degeneration, and neurodegenerative diseases [1]. Aging is a complex biological process and the process is modulated by multiple intrinsic and extrinsic factors such as hereditary, environmental, dietary, lifestyle, and stochastic factors throughout a person's lifetime [2]. Epigenetic alterations during aging are associated with modifications of DNA and histone proteins which straight affect chromatin structure and then affect gene expression and genomic stability [3]. The damage of various cell components such as organelles, especially protein and DNA damage, has been proposed as a factor causing aging on the cellular level [4]. Many changes of gene expression in the process of aging have been examined by some surveys based on the central dogma. And a number of pathways including the insulin/IGF-1 and TOR pathways and key factors including sirtuins and AMP kinase have been identified to regulate the aging process [5].

Moxibustion has been used for thousands of years in China and other Asian countries. Generally, moxibustion exerts its healing effects by burning the moxa herb* (Artemisia vulgaris)* on or above the skin at acupuncture points to warm the points [6]. Current studies have confirmed that moxibustion can efficiently control inflammation in bowel by regulating physiological balance at multiple links and multiple targets in the body with the low adverse reactions, low recurrence, and good long-term efficacy. Therefore, moxibustion has been applied diffusely [7]. It is reported that moxibustion can treat more than 300 kinds of diseases including malposition, diarrhea, colitis, urinary incontinence, dysmenorrhea, knee osteoarthritis, temporomandibular joint disturbance syndrome, soft tissue injury, heel pain, asthma, urinary retention, herpes zoster, and aging related problems [8]. In addition, moxibustion can improve immune function and physiological functions including cerebral blood flow in animals, which were suggested by the preclinical studies [9]. These findings exposed the potential value of moxibustion for giving humans some padding against aging in experimental research as well as practical application.

*Artemisia vulgaris* is a perennial weed growing wild and widespread in Asia, Europe, and North America. Normally, it abundantly grows in temperate and cold temperature zones and is known as mugwort or common wormwood [10].* Artemisia vulgaris* is being used for antihelminth, anticorrosion, and antispasm and used as a tonic for vital organs and in all kinds of disorders in traditional herbal medicine [11]. The chemical compositions of* Artemisia vulgaris*, including the essential oil (1,8-cineole, *β*-thujone, caryophyllene, germacrene D, and camphor), phenolic and flavonoid compounds (isoquercitrin, quercitrin, quercetin, luteolin, and kaempferol), hydroxycinnamic acids (gentisic, caffeic, p-coumaric, and ferulic acids), and some quinic acid derivatives such as 3-caffeoylquinic, chlorogenic, 5-feruloylquinic, 3,4-dicaffeoylquinic, 3,5-dicaffeoylquinic, 1,5-dicaffeoylquinic, 1,3-dicaffeoylquinic, 1,4-dicaffeoylquinic, and 4,5-dicaffeoylquinic acids, have been widely reported [12].

Digital gene expression (DGE) is a tag-based transcriptome sequencing method. DGE can be used to analyze quantitative gene expression and compare expression profiles without potential deviations in order to achieve sensitive and accurate transcriptome analysis [13]. DGE technology has been used more commonly in examining the differences of transcriptional responses in different tissues and organs. This is because DGE can identify millions of differentially expressed genes before prior annotation and the organisms without much genetic information are also allowed to be analyzed by DGE [14].

In the present study, RNA sequencing technology was used to evaluate the mechanisms that moxibustion functioned on aged mice. Based on the theory of traditional Chinese medicine about “the spleen being the foundation of acquired constitution,” only spleen samples were examined in DGE analysis. Differentially expressed genes in response to moxibustion were identified by assembling and annotating the transcriptome sequences which were identified in the spleen samples and analyzing the gene expression profiles.

## 2. Materials and Methods

### 2.1. Animals

Sixteen Institute of Cancer Research (ICR) female mice (19 months old) weighting 45 ± 5 g were purchased from the Beijing Vital River Laboratory Animal Technology Co., Ltd. (Beijing, China, Certificate number: SCXK (Beijing) 2012-0001). All the mice were housed in standard laboratory conditions (22°C ± 2°C, 50–60% indoor humidity) under a 12 h light–dark cycle (lights on from 8:00 am to 8:00 pm) and fed common chow. The experimental procedures were performed in strict accordance with the National Institutes of Health Guide for the Care and Use of Laboratory Animals. And all animal experiments in this study were approved by the Animal Ethics Committee of Beijing University of Chinese Medicine.

### 2.2. Moxibustion Intervention

Sixteen aged mice were randomly divided into the control and moxibustion groups (*n* = 8 mice per group). The normal mice were used as control group. The Guanyuan acupoint (RN4) was selected for treatment with moxibustion. The used moxa sticks (diameter 0.5 cm, length 20 cm) were provided by Nanyang Hanyi Moxibustion Technology Development Co., Ltd. (Henan, China). The mice were immobilized in fixators and the moxa stick was ignited and placed 1 cm above RN4 acupoint for 10 min to perform moxibustion. The mice in control group were immobilized for 10 min using the same method without moxibustion intervention. The treatment of moxibustion was carried out once in two days for a total of 60 days.

### 2.3. Sample Collection

After 60 days of moxibustion treatment, 6 healthier mice were selected from two groups (*n* = 3 mice per group). They were anesthetized with chloral hydrate and spleen specimens were collected. The obtained spleen samples were rapidly frozen in liquid nitrogen and stored at −80°C until RNA extraction.

### 2.4. RNA Extraction and Qualification

Total RNA was isolated from the spleen samples using Trizol reagent (Invitrogen, Canada) according to the protocol provided by manufacturer. RNA degradation and contamination were monitored on 1% agarose gels. RNA purity was checked with a NanoPhotometer® spectrophotometer (IMPLEN, USA). RNA concentration was determined by a Qubit® RNA Assay Kit in Qubit 2.0 Fluorometer (Life Technologies, USA) and RNA integrity was evaluated using RNA Nano 6000 Assay Kit of the Bioanalyzer 2100 system (Agilent Technologies, USA).

### 2.5. Library Preparation for DGE Sequencing

Sequencing libraries were produced using NEBNext® Ultra™ RNA Library Prep Kit (Illumina, USA) according to the recommendations of manufacturer. Three micrograms of RNA was used as the input material to sequence. Briefly, mRNA was purified from total RNA and fragmentation was carried out. Then first strand cDNA was synthesized with random hexamer primer and M-MuLV Reverse Transcriptase and second strand cDNA was subsequently synthesized. Following this, NEBNext Adaptor with hairpin loop structure was ligated. cDNA fragments of preferentially 150~200 bp in length were selected by purifying the library fragments using AMPure XP system (Beckman Coulter, USA). Then PCR was carried out and library quality was evaluated on the Agilent Bioanalyzer 2100 system.

### 2.6. Quality Control and Mapping Analyses

Raw reads of FASTQ format were processed with in-house Perl scripts. In this step, reads containing adapter sequences and ploy-N and low quality reads were removed from raw data to clean data, followed by calculating the Q20, Q30, and GC content of the clean data. The clean data with high quality were used for all the downstream analyses.

Bowtie v2.0.6 was used to build index of the reference genome and single-end clean reads were aligned to the reference genome through TopHat v2.0.9 [15]. TopHat can create a database of splice junctions on the basis of the gene model annotation file and thus generate a better mapping result than other nonsplice mapping tools. Therefore, TopHat was selected as the mapping tool.

### 2.7. Quantification of Gene Expression Level and Differential Expression Analysis

The number of reads mapped to each gene was counted using HTSeq v0.6.1 [16]. Then each gene's FPKM (fragments per kilobase of exon model per million mapped reads) value was calculated on the basis of the length of the gene and read count mapped to this gene. The effect of sequencing depth and gene length for the reads count was considered simultaneously by FPKM which is more accurate than RPKM (reads per kilobase of exon model per million mapped reads) in data processing of paired-end sequencing.

Before analysis of differential gene expression, edgeR program package was used to adjust the read counts for each sequenced library through one scaling normalized factor. The DESeq R package (1.12.0) was used to carry out the differential expression analysis of two groups [17]. There were three biological replicates per groups. The resulting *P* values were adjusted according to the Benjamini-Hochberg procedure to control the false discovery rate [18]. Genes with an adjusted *P* value < 0.05 and |log⁡2(fold  change)| > 1 found by DESeq were considered to be of differential expression.

### 2.8. GO and Pathway Analysis

In order to investigate the biological process, cellular component, and molecular function of differentially expressed genes, GO enrichment analysis was carried out by the GOseq R package [19]. Functional categories were enriched within genes and GO terms with *P* value < 0.05 were considered significantly enriched by differential expressed genes.

Kyoto Encyclopedia of Genes and Genomes (KEGG) database was used for pathway analysis of differential expression genes by the KOBAS software [20]. KEGG is the most commonly used database resource to understand high-level functions and utilities of the biological system, including the cell, the organism, and the ecosystem (http://www.genome.jp/kegg/).

## 3. Results

### 3.1. Analysis of DGE Libraries

Six DGE libraries were sequenced from 6 independent samples of female mice with or without moxibustion treatment to constitute a complete quantitative and qualitative gene expression response profile. Three individual samples marked as C1, C2, and C3 were included for the control group and another 3 individual samples marked as M1, M2, and M3 were included for the moxibustion group. The main characteristics of libraries were summarized and presented in Table 1. Approximately 26.83, 22.68, 21.59, 20.01, 25.58, and 27.05 million raw reads were obtained from the C1, C2, C3, M1, M2, and M3 libraries, respectively. And more than 96% of raw reads were clean reads in each library.

### 3.2. Mapping Reads to the Transcriptome

The clean reads from the six libraries were aligned to the reference genome to establish gene expression profiles. More than 84% of the clean reads mapped to the reference genome (Table 2). Less than 6% of the clean reads were multiple mapped reads which primarily came from the rRNA and intergenic regions [21]. The control group showed 21,033,084 (81.12%), 17,676,063 (80.62%), and 16,966,616 (81.54%) reads which were uniquely mapped to the reference genome, although the moxibustion group showed 16,552,756 (81.62%), 20,152,283 (81.63%), and 21,674,792 (82.99%) reads from M1, M2, and M3 libraries, respectively, uniquely mapped to the reference genome.

### 3.3. Analysis of Differential Gene Expression

**Figure 1:**
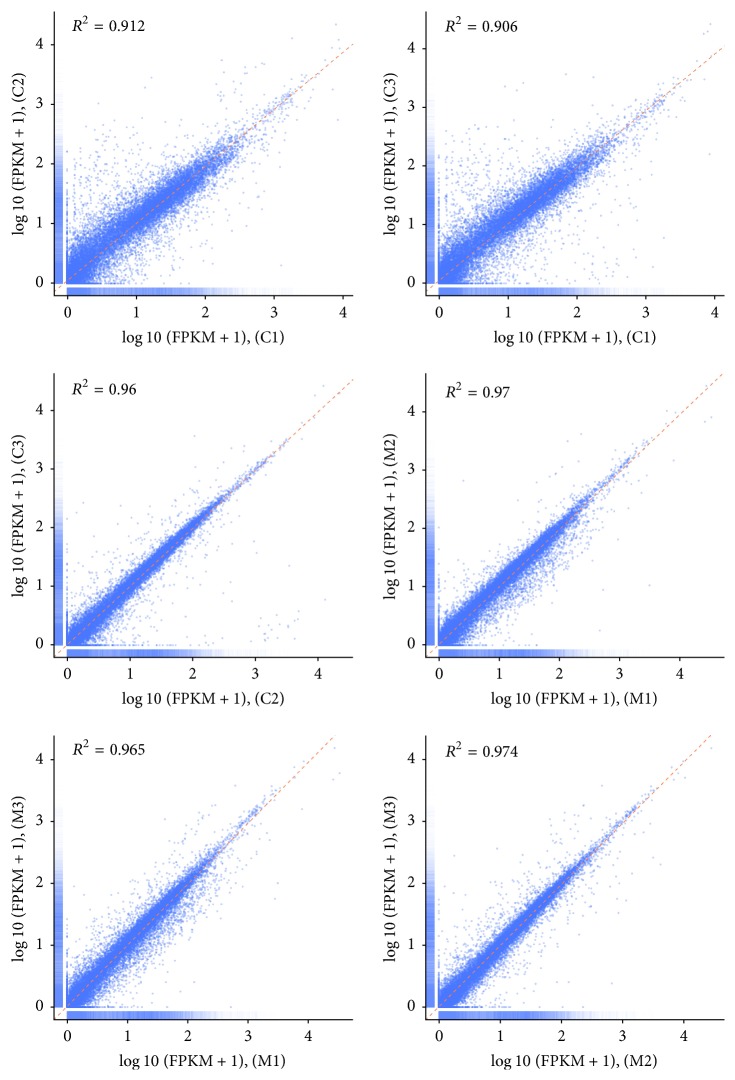
The correlation of FPKM values of all genes between the two replicates of control or moxibustion group. C1, C2, and C3: control group; M1, M2, and M3: moxibustion group. The logarithmic FPKM + 1 values of every gene in the two replicates were assigned as coordinate values of two axes.

The number of unambiguous clean tags for each gene was calculated in different FPKM value intervals for analysis of gene expression. Results showed that most FPKM of reads were between 0 and 1 (Table 3). Both of control group and moxibustion group showed significant and high correlation between any two replicates. The correlation coefficients were 0.912, 0.906, 0.96, 0.97, 0.965, and 0.974, respectively. Scatter diagrams were presented in Figure 1. The logarithmic FPKM +1 values were assigned as coordinate values of two axes and all data points exhibited a distribution in the region of the diagonal. An adjusted *P* value < 0.05 and |log⁡2(fold  change)| > 1 were chosen as the cutoff criteria to identify differentially expressed genes. Consequently, only 5 differentially expressed genes were eligible, including 4 upregulated and 1 downregulated genes (Table 4).

### 3.4. GO and KEGG Enrichment Analysis of Differentially Expressed Genes

**Figure 2:**
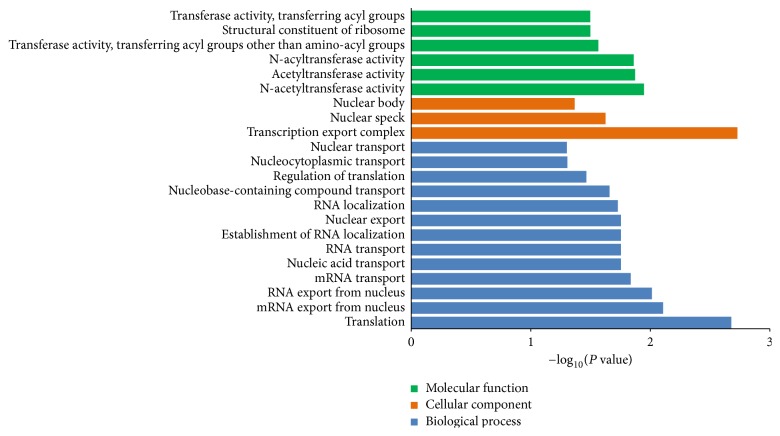
GO functional enrichment analysis. The differentially expressed genes between control and moxibustion groups were classified based on gene ontology.

Gene ontology of differentially expressed genes was analyzed based on biological process, cellular component, and molecular function. Twenty-two GO terms were significantly regulated by the expressed genes. The highly enriched GO terms in biological process, cellular component, and molecular function included translation, mRNA export from nucleus, mRNA transport, transcription export complex, nuclear speck, nuclear body, acetyltransferase activity, N-acyltransferase activity, and structural constituent of ribosome. The GO analysis results of differentially expressed genes are shown in Figure 2.

**Figure 3:**
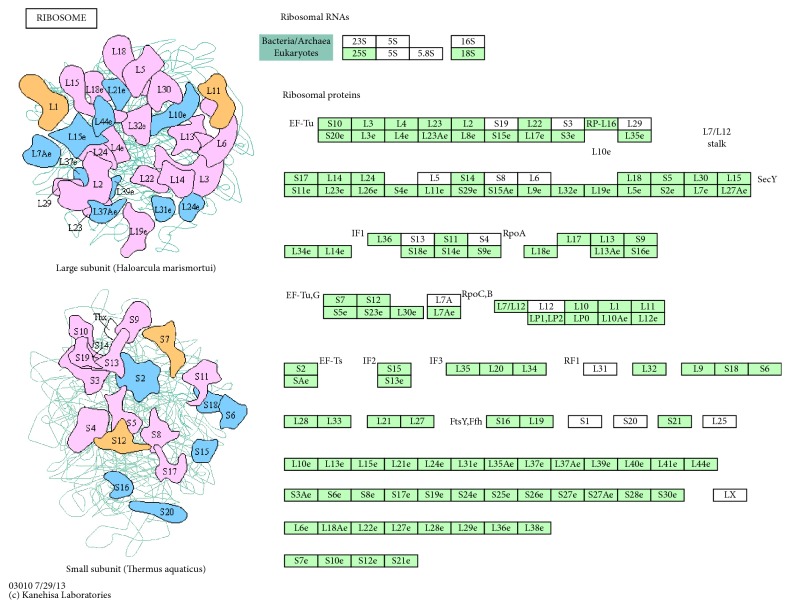
Pathway map of ribosome in KEGG.

The pathway significantly (corrected *P* value < 0.05) affected by differentially expressed genes was enriched through KEGG pathway analysis. Ribosome (corrected *P* value = 0.0059) was the most significantly enriched KEGG pathway term (Figure 3).

## 4. Discussion

In the present study, the female mice were treated with moxibustion and transcriptome analysis was carried out by using digital gene expression profiling. We investigated the influence of moxibustion on aged mice. It is believed that moxibustion can increase the generation of protective proteins including heat shock protein 70 (HSP70) and activate heat-sensitive neural release of nitric oxide to exert its efficacy [22]. According to biomedical theories, various pathways including elimination of free radical damage and regulation of immunity, neuroendocrine system, lipid metabolism, rheological characteristics of blood flow, trace elements, telomerase, and carbonyl poisoning are supposed to be the mechanisms by which moxibustion impact aging processes [23]. However, the mechanism by which moxibustion affects the genes expression of aged mice was still unknown. Therefore, transcriptome sequencing was carried out and compared for each sample.

The transcriptomes of spleen samples obtained from aged ICR mice given moxibustion for 60 days were sequenced to reveal the molecular mechanisms of moxibustion. In total, almost 145 million raw reads were obtained. Among them, approximately 140 million (96.55%) were clean reads. Five differentially expressed genes with an adjusted *P* value < 0.05 and |log⁡2(fold  change)| > 1 were identified between the control and moxibustion groups. They were Gm6563, Gm8116, Rps26-ps1, Nat8f4, and Igkv3-12.

In the light of GO enrichment analysis, the differentially expressed genes were enriched in molecular function, cellular components, and biological processes. According to the GO classifications, the differentially expressed genes were involved in structural constituent of ribosome, nucleus, mRNA export, mRNA transport, and translation. Results suggested that moxibustion plays a role in aged mice by influencing molecular function including acetyltransferase activity, n-acyltransferase activity, and structural constituent of ribosome. Moxibustion also affects cellular component including transcription export complex, nuclear speck, and nuclear body and biological process, such as translation, mRNA transport, and nuclear export. MRNA is released from nuclei and the number obviously decreases with age, which results from an impairment of polyadenylation of mRNA, hnRNA processing, release of mRNA from nuclear matrix, and translocations of mRNA from nuclear to cytoplasmic compartment [24]. Therefore, the level of mRNA released from nuclei is low in aged mice. But, this situation might be improved by moxibustion. It has been found that normal aging is connected with the exceptional enhancement in the generation and maturation of many mRNAs in human peripheral blood leukocytes [25]. A good deal of evidence proves that mRNA translation plays an important role in modulating aging. Studies have shown that lifespan can be increased by interventions resulting in decreased mRNA translation. Besides that, mRNA translation also affects various aging related processes and impacts on multiple longevity pathways [26]. Although the potential mechanisms for such regulation are still a mystery, it is definitely important to give the strong connection between mRNA translation and aging in different species [27]. In the present study, the functions of moxibustion target involved mRNA translation, which suggested that moxibustion might affect mRNA translation of aged mice. In addition, nuclear body, nuclear speck, and transcription export complex were the results of GO enrichment analysis in cellular component. Intriguingly, some evidence shows that nucleoli have additional functions connected with the cell cycle, cellular aging, signal recognition particle biosynthesis, small RNA processing, and mRNA transport [28]. Cellular aging is generally related to the instability of the nuclear and mitochondrial genomes and oxidative protein damage. That may directly lead to changes in transcription factors and chromatin structure and function. These changes gradually occur with aging and make for alterations of nuclear gene expression, mRNA stability, or both [29]. The finding of lamin A-dependent nuclear defects in human physiological aging further evidenced the correlation between nuclear envelope alterations and the process of aging [30]. Therefore, moxibustion may have a potential activity in antiaging because of intervention in molecular function, cellular components, and biological processes.

KEGG pathway analysis of the 5 differentially expressed genes was completed and result exhibited that a KEGG pathway named ribosome was the most significantly enriched. The ribosome, the integral but primarily passive participant in the synthesis of proteins across all kingdoms of life, is one of life's most ancient molecular machines. The ribosome has been considered as the decoder of the genome with high precision in the flow of biological information from mRNA to protein [31, 32]. It has been confirmed that the ribosome is huge machinery referred to translation of the genetic code into proteins and is one of the major targets of inhibitors for protein synthesis [33]. It revealed that moxibustion might function on aged mice by affecting protein synthesis through ribosome. There is increasing evidence revealing that the human pathological conditions characterized by an upregulated ribosome biogenesis can increase the risk of tumorigenesis [34]. Moxibustion may decrease the risk of tumorigenesis in aged mice by inhibiting the upregulation of ribosome biogenesis. Disturbances of ribosome homeostasis and hematopoietic dysfunction can be disproportionately caused by haploinsufficiencies of specific ribosomal proteins and defects in ribosome biogenesis [35]. The changes of morphology and function in the nucleolus were widely observed in cancer tissues, which have been indicated by the available evidence. These changes were caused by both the raised demand for ribosome biogenesis (the characteristic of proliferating cells) and the changes in the mechanisms which control cell proliferation [36]. The mRNA that carries the message of gene from the DNA leaves the nucleus to move to a ribosome in production of the particular protein. The ribosome moves along the mRNA for translating a ribonucleotide sequence into a corresponding amino acid sequence that constitutes the protein. Therefore, moxibustion may intervene in mRNA export and transport by ribosome pathway. According to the results of GO and KEGG enrichment analysis of differentially expressed genes, it was speculated that moxibustion functioned on aged mice by intervening in the biological process, such as translation, mRNA export from nucleus, and mRNA transport and pathways involved in the ribosome.

## 5. Conclusions

In the present study, we investigated the digital gene expression profiling of aged mice with or without moxibustion treatment and speculated the mechanisms of moxibustion in aged mice by analyzing the biological properties, molecular functions, and enriched pathways of differentially expressed genes. There were 5 significantly differentially expressed genes, namely, 4 upregulated genes and 1 downregulated gene. GO enrichment analysis exhibited that these genes were connected with structural constituent of ribosome, nucleus, mRNA transport, and translation. KEGG pathway analysis showed that they were enriched in the ribosome. According to the results of GO and KEGG enrichment analysis and considering the function of the ribosome, it was speculated that interventions to some biological process, such as translation, mRNA export from nucleus, and mRNA transport and pathways involved in the ribosome might be the mechanisms by which moxibustion functioned on aged mice.

## Figures and Tables

**Table 1:** Summary of sequencing analysis.

Item	C1	C2	C3	M1	M2	M3
Raw reads	26,827,940 (100%)	22,683,746 (100%)	21,586,308 (100%)	21,008,620 (100%)	25,584,692 (100%)	27,045,698 (100%)
Clean reads	25,927,296 (96.64%)	21,924,166 (96.65%)	20,808,688 (96.40%)	20,280,672 (96.54%)	24,686,554 (96.49%)	26,115,966 (96.56%)
Clean bases	3.89 G	3.29 G	3.12 G	3.04 G	3.70 G	3.92 G
Error rate	0.02%	0.02%	0.02%	0.02%	0.02%	0.02%
Q20	97.27%	97.24%	97.07%	97.2%	97.12%	97.19%
Q30	92.97%	92.92%	92.63%	92.86%	92.7%	92.84%
GC content	50.55%	50.32%	50.86%	50.59%	50.34%	50.34%

C1, C2, and C3: control group; M1, M2, and M3: moxibustion group. Q20: the percentage of bases with a Phred value > 20; Q30: the percentage of bases with a Phred value > 30.

**Table 2:** The data for the sequencing reads that mapped to the reference genome.

Mapping statistics	C1	C2	C3	M1	M2	M3
Total reads	25,927,296 (100%)	21,924,166 (100%)	20,808,688 (100%)	20,280,672 (100%)	24,686,554 (100%)	26,115,966 (100%)
Total mapped	22,227,826 (85.73%)	18,834,354 (85.91%)	17,754,405 (85.32%)	17,355,918 (85.58%)	20,950,545 (84.87%)	22,561,967 (86.39%)
Multiple mapped	1,194,742 (4.61%)	1,158,291 (5.28%)	787,789 (3.79%)	803,162 (3.96%)	798,262 (3.23%)	887,175 (3.4%)
Uniquely mapped	21,033,084 (81.12%)	17,676,063 (80.62%)	16,966,616 (81.54%)	16,552,756 (81.62%)	20,152,283 (81.63%)	21,674,792 (82.99%)
Reads mapped to “+”	10,510,392 (40.54%)	8,824,248 (40.25%)	8,466,976 (40.69%)	8,262,795 (40.74%)	10,073,579 (40.81%)	10,830,805 (41.47%)
Reads mapped to “−”	10,522,692 (40.59%)	8,851,815 (40.37%)	8,499,640 (40.85%)	8,289,961 (40.88%)	10,078,704 (40.83%)	10,843,987 (41.52%)
Nonsplice reads	11,296,626 (43.57%)	10,018,343 (45.7%)	10,020,095 (48.15%)	9,453,740 (46.61%)	11,610,290 (47.03%)	12,553,211 (48.07%)
Splice reads	9,736,458 (37.55%)	7,657,720 (34.93%)	6,946,521 (33.38%)	7,099,016 (35%)	8,541,993 (34.6%)	9,121,581 (34.93%)

C1, C2, and C3: control group; M1, M2, and M3: moxibustion group. “+” refers to the sense strand, and “−” refers to the antisense strand.

**Table 3:** Number of genes at different expression levels.

FPKM interval	0~1	1~3	3~15	15~60	>60
C1	27263 (67.21%)	2333 (5.75%)	4482 (11.05%)	4403 (10.85%)	2081 (5.13%)
C2	26185 (64.56%)	2510 (6.19%)	4951 (12.21%)	4938 (12.17%)	1978 (4.88%)
C3	26253 (64.72%)	2447 (6.03%)	4742 (11.69%)	4976 (12.27%)	2144 (5.29%)
M1	26524 (65.39%)	2410 (5.94%)	4710 (11.61%)	4808 (11.85%)	2110 (5.20%)
M2	26280 (64.79%)	2437 (6.01%)	4855 (11.97%)	4894 (12.07%)	2096 (5.17%)
M3	26317 (64.88%)	2505 (6.18%)	4724 (11.65%)	4904 (12.09%)	2112 (5.21%)

**Table 4:** List of all the differentially expressed genes between control and moxibustion group.

Gene ID	Gene name	Read count M	Read count C	Log 2 fold change	*P* value	*P*-adjusted	Regulated	Gene annotation
ENSMUSG00000051255	Gm6563	17.35	0.11	7.32	5.76E − 07	0.01	Up	SAP domain

ENSMUSG00000059422	Gm8116	1.35	22.08	−4.03	4.26E − 06	0.02	Down	-

ENSMUSG00000059775	Rps26-ps1	382.59	0.71	9.07	1.85E − 37	4.67E − 33	Up	Ribosomal protein S26e

ENSMUSG00000068299	Nat8f4	54.42	8.79	2.63	3.26E − 06	0.02	Up	GNAT domain, Acyl-CoA N-acyltransferase

ENSMUSG00000094117	Igkv3-12	576.18	147.63	1.96	2.25E − 06	0.02	Up	Immunoglobulin subtype, immunoglobulin V-set domain, immunoglobulin-like domain
